# Supplementary material for: Small protein Cgl2215 enhances phenolic tolerance by promoting MytA activity in Corynebacterium glutamicum
Source: Stress Biol. 2022 Nov 22;2(1):49. doi: 10.1007/s44154-022-00071-0 (PMC10441969; doi:10.1007/s44154-022-00071-0)
Supplement: Supplementary file 1 — Additional file 1: Fig. S1 Growth curve of C. glutamicum wild-type strain RES167 in MS medium supplemented with phenol (4 mM), ferulic acid (3 mM), or vanillin (3 mM). Fig. S2 Growth curves of C. glutamicum WT(vector), Δcgl2215(vector) and Δcgl2215(cgl2215) strains in LB broth. Table S1 Information of Cgl2215 homologs from non-redundant protein database. Table S2 Bacterial strains and plasmids used in this study. Table S3 Primers used in this study. [file 44154_2022_71_MOESM1_ESM.docx]

**Small protein Cgl2215 enhances phenolic tolerance by promoting MytA activity in *Corynebacterium glutamicum***

Huawei Gu^1#^, Xinwei Hao^1#^, Ruirui Liu^1^, Zhenkun Shi^1^, Zehua Zhao^1^, Fu Chen^1^, Wenqiang Wang^1^, Yao Wang^1^, Xihui Shen^1*^

^1^ State Key Laboratory of Crop Stress Biology for Arid Areas, Shaanxi Key Laboratory of Agricultural and Environmental Microbiology, College of Life Sciences, Northwest A&F University, Yangling, Shaanxi, China.

^*^ Correspondence: Xihui Shen, [xihuishen@nwsuaf.edu.cn](mailto:xihuishen@nwsuaf.edu.cn), ORCID 0000-0001-6867-8887

^#^ Contributed equally

# Abstract

*Corynebacterium glutamicum* is a promising chassis microorganism for the bioconversion of lignocellulosic biomass owing to its good tolerance and degradation of the inhibitors generated in lignocellulosic pretreatments. Among the identified proteins encoded by genes within the *C. glutamicum* genome, nearly 400 are still functionally unknown. Based on previous transcriptome analysis, we found that the hypothetical protein gene *cgl2215* was highly upregulated in response to phenol, ferulic acid, and vanillin stress. The *cgl2215* deletion mutant was shown to be more sensitive than the parental strain to phenolic compounds as well as other environmental factors such as heat, ethanol, and oxidative stresses. Cgl2215 interacts with *C. glutamicum* mycoloyltransferase A (MytA) and enhances its *in vitro* esterase activity. Sensitivity assays of the Δ*mytA* and Δ*cgl2215*Δ*mytA* mutants in response to phenolic stress established that the role of Cgl2215 in phenolic tolerance was mediated by MytA. Furthermore, transmission electron microscopy (TEM) results showed that *cgl2215* and *mytA* deletion both led to defects in the cell envelope structure of *C. glutamicum*, especially in the outer layer (OL) and electron-transparent layer (ETL)*.* Collectively, these results indicate that Cgl2215 can enhance MytA activity and affect the cell envelope structure by directly interacting with MytA, thus playing an important role in resisting phenolic and other environmental stresses.

**Key** **words:** *Corynebacterium glutamicum*; tolerance; phenolic compounds; MytA; mycoloyltransferase; cell envelope

## Introduction

Lignocellulose, the most abundant renewable resource on Earth, is a reliable source of raw materials for the biorefinery industry. In recent decades, microbial fermentation techniques using cheap and waste lignocellulosic raw materials such as corn cob, bagasse, and straw have become a research focus (Devi et al., 2022; Zhao et al., 2022). This bioconversion process requires a pretreatment step to break the crystal structure of cellulose and hydrolyze the lignocellulosic biomass into fermentable sugars (Capolupo and Faraco, 2016). However, several inhibitory substances are also produced during hydrolysis, including weak acids, phenolic compounds, and furan aldehydes, which can significantly inhibit the growth of microorganisms and affect the synthesis of target metabolites through microbial fermentation. The inhibitors in lignocellulose hydrolysate can be directly removed using detoxification chemicals, such as peroxidase, lime, ion exchange resin, or activated carbon (Guo et al., 2022). However, the removal steps lead to the loss of reducing sugar and require more equipment and a complex fermentation process, which greatly increases the fermentation cost.

Another option to reduce the impact of inhibitory substances on the fermentation process is to improve the tolerance or degradation ability of industrial strains to these inhibitors using methods such as strain domestication, random mutagenesis, or genetic engineering. *C. glutamicum* is an important industrial microorganism that can be used to produce amino acids, nucleotides, vitamins, recombinant proteins, and various value-added chemicals (Becker et al., 2018; Wolf et al., 2021). Recent studies have found that *C. glutamicum* has high tolerance and good degradation ability for lignin-derived aromatic compounds (for example, phenol, benzoate, phenylacetic acid, 4-cresol, ferulic acid, and vanillin), and can utilize some phenolic compounds as sole carbon and energy sources for growth and metabolism (Rodriguez et al., 2021; Wang et al., 2018). These characteristics give *C. glutamicum* unique advantages in the utilization of lignocellulose and it has become one of the most attractive and potential microorganisms to produce various value-added chemicals from renewable biomass resources. However, research on the tolerance mechanism of *C. glutamicum* to lignocellulose-derived inhibitors remains limited.

To systematically study the degradation and tolerance mechanisms of phenolic compounds and their relationship with other metabolic pathways in *C. glutamicum*, we previously assessed the response of *C. glutamicum* to ferulic acid, phenol, and vanillin stress through transcription levels using microarray experiments (Chen et al., 2016; 2017). Under the stress of different phenolic compounds, *C. glutamicum* improves its tolerance by directly degrading inhibitors, inducing the expression of heat shock and chaperone proteins, activating the intracellular SOS response, and regulating the expression of cell wall and membrane proteins. There were 140 differentially expressed genes (73 upregulated and 67 downregulated) under the three phenolic stress conditions, most of which mainly encoded proteins related to carbon source utilization and cell membrane structure (Chen, 2017). In addition to these known or putative functional genes, we also found that a gene (*cgl2215*) encoding a hypothetical small protein was upregulated at high levels under these stress conditions. We hypothesize that this gene may have an important effect on the tolerance of *C. glutamicum* to phenolic compounds and aimed to further investigate how this gene functions.

In this study, we evaluated the effect of *cgl2215* deletion on the tolerance of *C. glutamicum* to phenolic compounds and other stress conditions. The protein interacting with Cgl2215 in *C. glutamicum* was screened and identified. The effect of Cgl2215 on the function of interacting proteins was further analyzed. These results revealed the function of Cgl2215 and provided insights into the stress tolerance mechanism of *C. glutamicum*.

## Results

### Significant accumulation of *cgl2215* transcript under phenolic stress

Based on the microarray data, we first analyzed the transcription levels of *cgl2215* in *C. glutamicum* in response to various phenolic compounds, including phenol, ferulic acid, and vanillin. The growth curves of *C. glutamicum* wild-type strain RES167 in the presence or absence of 4 mM phenol, 3 mM ferulic acid, or 3 mM vanillin were displayed in Fig.S1. Quantitative reverse transcription-polymerase chain reaction (qRT-PCR) was performed using the total cellular RNA samples prepared from cultures grown at the late exponential phase (10 h). The 16S rRNA gene was used as an internal control to calculate the relative expression levels of *cgl2215*. As shown in Fig. 1A, the expression levels of *cgl2215* from *C. glutamicum* exposed to phenol, ferulic acid, and vanillin increased by 11.7-, 21.7-, and 16.0- fold, respectively.

### ****Bioinformatics analysis of Cgl2215 protein****

The *cgl2215* gene is 213 bp long and encodes a hypothetical small protein without any known functions or protein family classification. Computer analysis indicated that the theoretical molecular mass and isoelectric point of Cgl2215 were 8.2 kDa and 6.83, respectively. A BLAST search against the non-redundant protein sequence database returned eight homologs of Cgl2215 with high identities (>70%), all of which were from the genus *Corynebacterium* (Table S1). Multiple sequence alignment and phylogenetic tree analysis were performed based on the amino acid sequences of Cgl2215 and its homologs (Figs. 1B and C). These results show that Cgl2215 homologs are highly conserved and have only been found in several *Corynebacterium* species.

### Positive role of Cgl2215 on combating phenolic stress in *C. glutamicum*

To further study the role of Cgl2215 in response to phenolic compounds, the Δ*cgl2215* mutant and complemented strain Δ*cgl2215*(*cgl2215*) were constructed. To exclude the effects of the heterologous plasmid, the pXMJ19 vector was transferred into the *C. glutamicum* RES167 and Δ*cgl2215* mutants, generating the parental strains WT(vector) and Δ*cgl2215*(vector), respectively. *cgl2215* deletion and complementation did not notably affect the growth of *C. glutamicum* in Luria-Bertani (LB) broth (Fig. S2). However, when cultured with phenolic compounds, the Δ*cgl2215*(vector) mutant was showed more sensitive to phenolic compounds than the parental strain was (Fig. 2A). When *cgl2215* was complemented, the cell growth returned to nearly the same level as that of the parental strain. After 30 min of incubation with phenolic compounds, the survival rates of the Δ*cgl2215*(vector) mutant were also significantly lower than that of the parental strain, whereas the survival rates of the Δ*cgl2215*(*cgl2215*) strain were not significantly different from those of the parental strain (Fig. 2B). The role of Cgl2215 in response to other environmental stressors, including H_2_O_2_, ethanol, and heat, was also tested. The mutant lacking the *cgl2215* gene was more sensitive to these stressors than the parental strain WT(vector) and Δ*cgl2215*(*cgl2215*) strain (Fig. 2C), indicating a positive role for Cgl2215 in *C. glutamicum* in response to multiple environmental stresses.

### Cgl2215 interacts with *C. glutamicum* mycoloyltransferase A (MytA)

Recombinant GST-Cgl2215 protein was used as the bait protein incubated with *C. glutamicum* lysate to screen for target proteins that may interact with Cgl2215. After removing non-specific binding proteins, the proteins captured by magnetic beads were separated using sodium dodecyl-sulfate polyacrylamide gel electrophoresis (SDS-PAGE) and stained with silver. We found that the proteins of approximately 70 kDa were specifically captured by GST-Cgl2215-loaded magnetic beads (Fig. 3A). Mass spectrometry analysis showed that the most abundant protein of the 70 kDa band encodes *C. glutamicum* MytA (Cgl2875). Glutathione S-transferase (GST) pull-down and bacterial two-hybrid assays were performed to verify the interactions between MytA and Cgl2215. Western blotting results showed that a specific interaction between MytA and GST-Cgl2215 was observed (Fig. 3B). Only the *E. coli* BTH101 strain containing *cgl2215* and *mytA* genes showed a blue colony phenotype and significantly higher β-galactosidase activity than the control strains (Fig. 3C). These results indicate that Cgl2215 interacts with MytA.

### MytA is involved in Cgl2215-mediated tolerance to phenolic compounds

The direct interaction between MytA and Cgl2215 prompted us to explore whether MytA plays a role in the resistance to phenolic stress. Based on qRT-PCR experimental results, high expression levels of *mytA* were also found in response to phenolic and other environmental stresses (Fig. 4A). The survival rates of the Δ*mytA*(vector) mutant under phenolic stress were significantly lower than those of the parental strain or complemented strain (Fig. 4B). These results indicate that MytA also has a significant effect on tolerance to phenolic stress in *C. glutamicum*. We constructed the *C. glutamicum* Δ*cgl2215*Δ*mytA* strain to determine the relationship between MytA and Cgl2215 in response to phenolic stress. As shown in Fig. 4C, the Δ*cgl2215*Δ*mytA* strain had a low survival rate (17.0%) in the sensitivity assay. Complementation of either *mytA* or *cgl2215* alone did not significantly improve its tolerance ability. However, complementation with both *cgl2215* and *mytA* significantly increased the survival rate of the strains under phenolic stress. Collectively, these results establish that the role of Cgl2215 in phenolic tolerance may be mediated by MytA.

### Cgl2215 enhances the *in vitro* esterase activity of MytA

MytA, also referred to as PS1 or CspA, is a major mycoloyltransferase in *C. glutamicum* and is involved in the transfer of mycolic acid residues onto the cell wall arabinogalactan and trehalose monomycolate (Puech et al., 2000; Takeshita et al., 2010).

The interaction between Cgl2215 and MytA led us to hypothesize that Cgl2215 directly affects the enzymatic activity of MytA. Prediction of the protein complex showed that Cgl2215 binds to the N-terminal catalytic domain of MytA (Fig. 5A). The *in vitro* esterase activity of MytA was further tested in the presence or absence of Cgl2215, by determining the release of *p*-nitrophenol (*p*-NP) from the synthetic substrate *p*-nitrophenyl palmitate (*p*-NPP). As shown in Fig. 5B, MytA exhibited esterase activity, whereas GST-Cgl2215 or GST did not. In the presence of GST-Cgl2215 with a 1:1 ratio of GST-Cgl2215:MytA, the esterase activity of MytA increased 1.6-fold, and with an increase in the ratio of GST-Cgl2215:MytA, the esterase activity of MytA was further increased. These results indicate that Cgl2215 may promote MytA esterase activity by directly binding to the N-terminal domain of MytA.

### Effect of *cgl2215* deletion on the *C. glutamicum* cell envelope

Based on the essential role of MytA in cell envelope biosynthesis of *C. glutamicum* and the promoting effect of Cgl2215 on MytA esterase activity, we further verified the effects of *cgl2215* deletion on the cell envelope of *C. glutamicum* using transmission electron microscopy (TEM). In accordance with previous reports (Bayan et al., 2003; Kacem et al., 2004), the cell envelope structure of the *C. glutamicum* parental strain examined using TEM is composed of four different layers: an outer layer (OL), a thin electron-transparent layer (ETL), a thick electron-dense layer (EDL), and a plasma membrane (PM) (Fig. 5C). Compared with the parental strain, the Δ*cgl2215*(vector) mutant presented a thinner cell envelope without a clear OL, but a dispersive ETL, which was similar to the Δ*mytA*(vector) mutant. Complementation of *cgl2215* or *mytA* in the corresponding mutants fully restored the cell envelope structure. These results indicate that the deletion of either *cgl2215* or *mytA* lead to defects in the cell envelope structure in *C. glutamicum*, especially for the OL and ETL, which may be the main factor affecting the tolerance of strains to phenolic compounds.

## Discussion

Because of the good tolerance to the inhibitors generated in lignocellulosic pretreatments, *C. glutamicum* is a promising chassis microorganism for the bioconversion of lignocellulosic biomass (Lin et al., 2022). Among 2960 identified proteins within the *C. glutamicum* genome, nearly 400 proteins are functionally unknown. A thorough study of these hypothetical protein genes will clarify the genetic background of *C. glutamicum* cells and provide more references for improving the robustness of engineered *C. glutamicum* strains. Here, an uncharacterized small protein, Cgl2215, was selected to verify its role in phenolic tolerance based on our previous transcriptomic analysis of *C. glutamicum* in response to phenol, ferulic acid, and vanillin (Chen, 2017; Chen et al., 2016; 2017).

The inhibitory effects of lignin-derived phenolic compounds on fermentative microorganisms are closely related to their hydrophobicity and the presence of formyl, carboxyl, and hydroxyl groups. They can disintegrate cell membranes, resulting in the leakage of intracellular components, thereby affecting the intracellular metabolism of cells and cell survival (Kamimura et al., 2017). We found that deletion of the *cgl2215* gene resulted in a significant decline in the survival and growth ability of *C. glutamicum* in the presence of phenolic compounds (Figs. 2A and B). These results indicate that the unknown small protein Cgl2215 plays a crucial role in resistance to lignin-derived phenolic compounds. However, we failed to predict the possible biological functions of Cgl2215 based on amino acid sequence analysis, as we did not find any information through NCBI-conserved domain analysis and InterPro scan, with only a few homologs with high identities from the *Corynebacterium* genus discovered using BLAST and multi-sequence alignment (Figs.1B and C). This led us to hypothesize that Cgl2215 may perform specific functions in *Corynebacterium*.

To reveal the roles of Cgl2215 in *C. glutamicum*, we used the GST-pull down assay combined with mass spectrometry to screen target proteins in cell lysates and found that MytA interacts with Cgl2215 (Fig. 3). Some high G+C gram-positive bacteria such as *Corynebacterium* and *Mycobacterium* have a special cell envelope structure featuring a distinctive outer membrane, referred to as the mycomembrane, which is composed of mycolic acids covalently linked to a thick arabinogalactan-peptidoglycan polymer outside the plasma membrane (Marrakchi et al., 2014). MytA is a major mycoloyltransferase in *C. glutamicum* and is responsible for the synthesis of the mycomembrane backbone by transferring the mycolic acid residues onto trehalose monomycolate (TMM), forming trehalose dimycolate (TDM), or onto the terminal arabinose of arabinogalactan (Dautin et al., 2017; De Sousa-D'auria et al., 2003).

This unique mycomembrane structure is thought to be crucial for the structural organization of the cell envelope and functions as an outer permeability barrier to confer cells with high resistance to antibiotics, solutes, and host defense mechanisms (Lanéelle et al., 2013; Rahlwes et al., 2019). Previous study found that the Δ*mytA*Δ*mytB* mutant of *C. glutamicum* CGL2005 exhibited a growth defect at 30 ℃ and was completely devoid of the outermost layer (Kacem et al., 2004). The disruption of *mytA* in *C. glutamicum* ATCC13869 led to increased alkali sensitivity and susceptibility to ethambutol, penicillin, and rifampicin (Takeshita et al., 2010). Here, we found that *mytA* expression was induced in response to phenolic compounds, heat, ethanol, and oxidative stress (Fig. 4A). The disruption of *mytA* resulted in a decline in the survival rate with phenolic compounds and defects in the mycomembrane (Figs. 4B and 5C). These results support the important role of *C. glutamicum* MytA in mycomembrane formation and resistance to environmental stresses.

To determine the role of MytA in Cgl2215-mediated phenolic tolerance, we compared the sensitivity of the Δ*cgl2215*Δ*mytA* double mutant and its complemented strains to phenol and found that the survival rate was only restored by complementation with both *cgl2215* and *mytA* (Fig. 4C). These results suggest that the role of Cgl2215 in phenolic tolerance is closely related to that of MytA. To confirm the role of MytA in Cgl2215-mediated phenolic tolerance, molecular docking was performed. Cgl2215 was docked to the N-terminal catalytic domain of MytA (Fig. 5A). Based on the findings of this model, Cgl2215 may be bound near to the conserved catalytic residue H366 of MytA through hydrogen bonding. Thus, we examined the effect of Cgl2215 on the enzymatic activity of MytA and did find that Cgl2215 positively enhanced the *in vitro* esterase activity of MytA (Fig. 5B). In addition, similar to MytA, Cgl2215 affected mycomembrane formation (Fig. 5C). These results support the hypothesis that MytA mediates the role of Cgl2215 in phenolic tolerance. Cgl2215 may participate in phenolic tolerance by promoting MytA activity and affecting mycomembrane synthesis.

Microorganisms have developed extensive strategies to cope with environmental stresses over a long evolutionary period. Previous studies have revealed that *C. glutamicum* can resist phenolic stress through many methods, based on the regulation of intracellular metabolism (Ding et al., 2015; Liu et al., 2013; Shen et al., 2012; Wang et al., 2018; Zhou et al., 2019). However, studies on cell walls as the first barrier against phenolic and other environmental stresses are still limited. Our earlier transcriptome data showed that many genes related to the cell envelope are differentially regulated (Chen et al., 2016; 2017). Here, we revealed for the first time that a small protein, Cgl2215, can resist phenolic stress and other environmental stresses by participating in the formation of mycomembranes. Although Cgl2215 was initially found to be related to phenolic tolerance, we hypothesized that it would be a broad and non-specific tolerance mechanism for *C. glutamicum* because its target protein MytA is closely related to cell envelope biosynthesis.

Notably, Cgl2215 homologs were only found in a few *Corynebacterium* species, whereas MytA is conserved and widely occurring in *Corynebacterium* as well as in mycolic acid-containing bacteria. Whether Cgl2215 could interact with MytA homologs from other bacteria and affects their tolerance to environmental stresses? In addition, we also identified several Cgl2215-interacting proteins such as AccBC, Tkt and SdhA through mass spectrometry in GST pull-down screening experiment. Cgl2215 may also affect the central carbon metabolism by interacting with these enzymes in response to phenolic stress. These still need further study and the results will be of great significance and reference value for the construction of improved engineered strains.

In conclusion, a new uncharacterized small protein in *C. glutamicum*, Cgl2215, was identified with positive roles in tolerance to phenolic compounds, heat, ethanol, and oxidative stress. The mycoloyltransferase MytA is a target protein of Cgl2215 in *C. glutamicum*. Cgl2215 can enhance MytA activity and affect the integrity of the cell envelope structure by directly interacting with MytA, thereby resisting environmental stresses. These insights provide a new reference for the genetic modification of strains to improve their tolerance to environmental factors and efficiently produce value-added chemicals or biofuels from lignocellulosic biomass.

## Materials and methods

### Bacterial strains and growth conditions

*C. glutamicum* strains were cultured in LB broth or mineral salt (MS) medium (0.5 g/l KH_2_PO_4_, 1 g/l Na_2_HPO_4_·12H_2_O, 0.03 g/l MgSO_4_·7H_2_O, 1.07 g/l NH_4_Cl, and 0.05 g/l yeast extract; pH 8.4) at 30 °C. Brain heart infusion (BHI) broth supplemented with sorbitol (0.5 M) was used to prepare competent *C. glutamicum* cells. *E. coli* strains were grown at 37 °C in LB broth. The following antibiotics were added to the medium as necessary: kanamycin (50 μg/ml for *E. coli* and 25 μg/ml for *C. glutamicum*), chloramphenicol (20 μg/ml for *E. coli* and 10 μg/ml for *C. glutamicum*), nalidixic acid (40 μg/ml), and ampicillin (100 μg/ml). *C. glutamicum* RES167 was the parental strain of all derivatives used in this study. The bacterial strains and plasmids used in the present study are listed in Table S2.

### Bioinformatics analysis

ProtParam (http://web.expasy.org/protparam/) was used to compute the physical and chemical parameters of Cgl2215. BLAST was used for the homology search of Cgl2215 against the non-redundant protein sequence (nr) database. Proteins sharing more than 40% sequence identities and E-values less than 0.01 are referred to as Cgl2215 homologs. BLAST-aligned protein sequences were used for multiple sequence alignment using Clustal W, and a phylogenetic tree was constructed using the neighbor-joining method with MEGA 11 software (Tamura et al., 2021). The conserved domain database (CDD) (Marchler-Bauer et al., 2017) was used to identify the conserved domain present in Cgl2215. The AlphaFold-Multimer tool (Evans et al., 2021) was used to predict the complex structure of Cgl2215 and MytA. The interaction between proteins was analyzed based on the top-ranked model to calculate the distance between atoms (Cα) of the interacting residues and to determine the type of interaction.

### Construction of *C. glutamicum* mutants and complemented strains

The *C. glutamicum* mutants in this study were constructed using homologous recombination as previously described (Shen et al., 2005). The upstream and downstream homologous arm fragments of the target gene were amplified and inserted into the pK18*mobsacB* vector. Primers used in this study are listed in Table S3. The recombinant plasmid was transformed into the *C. glutamicum* parental strain through electroporation. The single crossover strains were screened on BHI plates supplemented with nalidixic acid and kanamycin, then grown overnight in LB broth without kanamycin, allowing a second crossover to occur. After appropriate dilution, the cultures were spread on plates containing nalidixic acid and 20% sucrose to obtain strains that lost the integrated *sacB*-containing plasmid. These colonies were further screened using plate culture for kanamycin-sensitive and nalidixic acid-resistant strains. After verification using PCR and DNA sequencing, a mutant strain was successfully constructed. For complementation experiments, gene fragments amplified using the genomic DNA of *C. glutamicum* as a template were ligated into the pXMJ19 vector and transferred into the mutant strain. Colonies grown on LB plates containing chloramphenicol and nalidixic acid were verified using PCR to obtain the complemented strain.

### RNA isolation and qRT-PCR

*C. glutamicum* cells cultured with or without 4 mM phenol, 3 mM ferulic acid, and 3 mM vanillin were collected through centrifugation. Total RNA was extracted using the TRIzol method. The FastKing RT Kit (with gDNase) (Tiangen, China) was used to synthesize cDNA according to the manufacturer’s instructions. Relative expression levels of target transcripts were determined using TransStart Green qPCR SuperMix (TransGen Biotech, China) and a real-time PCR system (Roche, Switzerland). 16S rRNA was used as an internal control, and the expression of target genes was calculated as relative fold values using the 2^-ΔΔCt^ method. Three biological replicates were assayed, and statistical analysis was performed using the Student’s t-test.

### Sensitivity assay

*C. glutamicum* strains cultured in the late log phase were collected, washed, and diluted 100-fold in MS medium. The stressors (phenol (50 mM), ferulic acid (8 mM), vanillin (50 mM), H_2_O_2_ (20 mM), or ethanol (1.72 M)) were added to the cells and incubated at 30 °C for 30 min. Cells not treated with stressors were used as controls. After treatment, the samples were diluted with appropriate gradient, plated onto LB plates, and cultured at 30 °C for an additional 2 d. Survival rates were calculated from the ratio of the number of colonies with treatment to those without treatment. All assays were performed in triplicate at least twice, and statistical analysis was performed using two-way ANOVA. Statistical significance was set at p < 0.05. The plate culture method was used to show the effects of phenolic stress on the growth of *C. glutamicum.* Fresh *C. glutamicum* culture (2.5 μl) prepared through serial dilution were dropped onto LB plates containing phenolic compounds (phenol (32 mM), ferulic acid (5 mM) or vanillin (20 mM)). After the samples were completely absorbed, the plates were transferred to a 30 °C incubator for 2 d. The plates were then observed and photographed.

### Protein expression and purification

*C. glutamicum* *cgl2215* and *mytA* gene fragments were amplified and ligated into the pGEX-6P-1 and pET-28a vectors, respectively. Recombinant expression vectors were transformed into *E. coli* BL21(DE3) cells for protein expression. The overnight cultured bacteria were inoculated in 300 ml fresh LB broth containing the corresponding antibiotics and incubated at 37 °C until an OD_600_ of approximately 0.3–0.4. After cooling, 0.5 mM IPTG was added to the culture for induction of protein expression at 26 ℃ for 8 h. The cells were washed, resuspended in 20 mM Tris-HCl buffer (pH 8.0), and lysed using ultrasonication. The supernatant was harvested using centrifugation and loaded onto a pre-equilibrated Ni-NTA or GST-Bind resin. Unbound proteins were removed by washing with five column volumes of washing buffer. Finally, elution buffer with 0.5 M imidazole or 10 mM glutathione (GSH) was used to elute the recombinant protein. The purity of the purified protein was tested using SDS-PAGE, and the protein concentration was determined using the Bradford protein assay according to the manufacturer’s instructions (Bio-Rad, USA).

### GST pull-down assay

To screen for potential proteins interacting with Cgl2215, a GST pull-down assay with cell lysates was performed as previously described (Si et al., 2017). Purified GST-Cgl2215 or GST protein (0.1 mg) was incubated with 30 μl GST magnetic beads at 4 ℃ for 2 h. After washing with Tris-HCl buffer (pH 8.0) containing 150 mM NaCl, the beads were incubated with the wild-type *C. glutamicum* lysates overnight at 4 °C. The supernatant was discarded and the beads were washed five times with TEN buffer (50 mM Tris-HCl, pH 8.0, 500 mM NaCl) to remove the unbound protein. Proteins bound to the beads were dissolved in SDS loading buffer for SDS-PAGE. After visualization using silver staining, specific single bands retained by the beads coated with GST-Cgl2215 were analyzed using liquid chromatography with tandem mass spectrometry (LC-MS/MS).

To validate the interaction between MytA and Cgl2215, His_6_-MytA and GST-Cgl2215 were coexpressed in *E. coli* BL21(DE3). BL21(pET28a-*mytA*), BL21(pET28a-*mytA*/pGEX-6P-1), and BL21(pET28a-*mytA*/pGEX-6P-1-*yezP*) strains were constructed as blank, negative, and unrelated protein controls, respectively. After the initial growth of these strains to an OD_600_ of 0.6, 1.0 mM IPTG was added to the culture for inducible expression. Bacterial cells were lysed using sonication. The supernatant collected using centrifugation was loaded onto the GST-binding resin. After washed with five-column volume of washing buffer, proteins retained on the resin were dissolved in SDS loading buffer and detected using western blotting.

### Bacterial two-hybrid analysis

The *cgl2215* and *mytA* DNA fragments were amplified and ligated into pUT25m and pKT18cm to obtain recombinant plasmids pUT18cm-*mytA* and pKT25m-*cgl2215*. The recombinant plasmids were then electrotransformed into competent *E. coli* BTH101 cells. The strain containing pKT25m-*cgl2215* and pKT18cm was used as a negative control, whereas the strain containing pKT25m-*zip* and pKT18cm-*zip* was used as a positive control. The color and the enzymatic activity assays were performed as previous described (Battesti and Bouveret, 2012), with several modifications. Briefly, for the color assay, 2 μl of each culture was dropped onto LB plates supplemented with 100 μg/ml ampicillin, 50 μg/ml kanamycin, 0.5 mM IPTG, and 30 μg/ml X-Gal. After incubation for 2 d at 30 ℃, the plates were imaged. For the β-galactosidase assay, bacterial cells were cultured overnight in 2 ml fresh LB medium (containing 100 μg/ml ampicillin, 50 μg/ml kanamycin, and 0.5 mM IPTG) at 30 ℃ until late-logarithmic phase. Fifty microliters of culture were transferred into a 1.5 ml centrifuge tube containing 420 μl Z-buffer, 20 μl trichloromethane, and 10 μl 0.1% SDS, mixed well, and incubated at 30 ℃ for 30 min. Then, 100 μl of 4 mg/ml 2-Nitrophenyl β-D-galactopyranoside (ONPG) substrate solution preheated to 30 ℃ was added to start the enzymatic reaction. A total of 250 μl of 1 M Na_2_CO_3_ was used to stop the reaction and the reaction time was recorded. The reaction solution (200 μl) was transferred into a 96-well flat-bottom microplate for OD_420_ and OD_550_ measurements. The relative β-galactosidase activity was calculated as follows: 1000×(OD_420_-1.75×OD_550_)/(reaction time(min)×OD_600_×V_bacterial suspension_(L)).

### MytA esterase activity assay

The esterase activity of MytA was assayed *in vitro* using synthetic substrates as previously described (Battesti and Bouveret, 2012), with several modification. Purified MytA was incubated with *p*-NPP as a substrate, and the enzymatic activity was determined by measuring the accumulation of *p*-NP over time. The reaction system contained 0.1 M TES (N-tris(hydroxymethyl)methyl-2-aminoethanesulfonicacid) (pH 7.5), 250 μM *p*-NPP, 13 mM NaCl, and 0.05% Triton X-100. MytA was used at the final concentration of 4.7 μM. The effects of Cgl2215 on MytA enzymatic activity were investigated by adding purified GST-Cgl2215 at different molar concentrations. To eliminate the possible influence of the GST tag on the results, GST only group, GST-Cgl2215 only group, and GST/MytA group were used as control simultaneously. After incubation at 37 ℃ for 6 h, the *p*-NP consumption was monitored by measuring the absorbance at 405 nm. The results are presented as the mean of the absorbance and standard error of the mean of at least three replicates. Statistical analysis was performed using ordinary one-way ANOVA. Statistical significance was set at p < 0.05.

### TEM

TEM was performed following a previously described procedure (Kacem et al., 2004), with some modifications. *C. glutamicum* cells harvested at the late-exponential phase were washed twice with 0.1 M phosphate-buffered saline (PBS; pH 7.4) and fixed overnight with precooled 2.5% glutaraldehyde at 4 °C. After washing with 0.1 M PBS, the cells were post-fixed with 1% osmium tetroxide for 2–3 h and dehydrated using a graded ethanol series (30%, 50%, 70%, 80%, 90%, and 100%; twice for 10 min each). The dehydrated cells were then embedded in LR white. The ultrathin sections were sliced using an ultramicrotome to a thickness of 50–70 mn and double-stained with uranyl acetate and lead citrate. For TEM observations, all images were captured using an HT7800 TEM (Hitachi, Japan).

## Abbreviations

BHI: Brain-heart infusion; *C. glutamicum*: *Corynebacterium glutamicum*; EDL: electron-dense layer; ETL: electron-transparent layer; IPTG: Isopropyl β-D-thiogalactoside; LB: Luria-Bertani; MS: mineral salt; ONPG: 2-Nitrophenyl β-D-galactopyranoside; OL: outer layer; *p*-NPP: *p*-Nitrophenyl palmitate; *p*-NP: *p*-nitrophenol; PM: plasma membrane; TEM: Transmission electron microscopy.

## Acknowledgements

We thank all colleagues involved in this work for their contributions to this article.

## Authors’ contributions

H.G. and X.S. conceived the project and designed the experiments. H.G., R.L., and X.H. performed the majority of experiments. H.G. performed the computational analyses and provided technical support. Z.Z., F.C., Z.S., Q.W., and Y.W. helped in strains construction, protein purification and interaction verification, and phenotypic experiments. H.G. wrote the paper. X.S. revised the manuscript and supervised the study. All authors read and approved the final manuscript.

## Funding

This work was supported by the grant of National Key R&D Program of China (2021YFA0909600 to X.S.), and the National Natural Science Foundation of China (31670053 to X.S., 32000022 to H.G.), China Postdoctoral Science Foundation (2019M663829 to H.G.) and Northwest A&F University starting research fund (Z1090219039 to H.G.).

## Availability of data and materials

All data generated or analyzed are available in the paper and online supplemental files.

## Declarations

### Ethics approval and consent to participate

Not applicable.

### Consent for publication

All authors agree to publish.

### Competing interests

The authors declare no compete of interests.

### Author details

^1^ State Key Laboratory of Crop Stress Biology for Arid Areas, Shaanxi Key Laboratory of Agricultural and Environmental Microbiology, College of Life Sciences, Northwest A&F University, Yangling, Shaanxi, China.

## Reference

Battesti A, Bouveret E (2012) The bacterial two-hybrid system based on adenylate cyclase reconstitution in *Escherichia coli*. Methods 58(4): 325-334. <https://doi.org/10.1016/j.ymeth.2012.07.018>

Bayan N, Houssin C, Chami M, Leblon G (2003) Mycomembrane and S-layer: two important structures of Corynebacterium glutamicum cell envelope with promising biotechnology applications. J Biotechnol 104(1-3): 55-67. <https://doi.org/10.1016/s0168-1656(03)00163-9>

Becker J, Rohles C M, Wittmann C (2018) Metabolically engineered *Corynebacterium glutamicum* for bio-based production of chemicals, fuels, materials, and healthcare products. Metab Eng 50: 122-141. <https://doi.org/10.1016/j.ymben.2018.07.008>

Capolupo L, Faraco V (2016) Green methods of lignocellulose pretreatment for biorefinery development. Appl Microbiol Biotechnol 100(22): 9451-9467. <https://doi.org/10.1007/s00253-016-7884-y>

Chen C, Pan J, Yang X, Guo C, Ding W, Si M, Zhang Y, Shen X, Wang Y (2016) Global Transcriptomic Analysis of the Response of *Corynebacterium glutamicum* to Vanillin. PLoS One 11(10): e0164955. <https://doi.org/10.1371/journal.pone.0164955>

Chen C (2017) Studies on the tolerance mechanism of *Corynebacterium glutamicum* to aromatic compounds. Dissertation, Northwest A＆F University

Chen C, Pan J, Yang X, Xiao H, Zhang Y, Si M, Shen X, Wang Y (2017) Global transcriptomic analysis of the response of *Corynebacterium glutamicum* to ferulic acid. Arch Microbiol 199(2): 325-334. <https://doi.org/10.1007/s00203-016-1306-5>

Dautin N, de Sousa-d'Auria C, Constantinesco-Becker F, Labarre C, Oberto J, Li de la Sierra-Gallay I, Dietrich C, Issa H, Houssin C, Bayan N (2017) Mycoloyltransferases: A large and major family of enzymes shaping the cell envelope of *Corynebacteriales*. Biochim Biophys Acta Gen Subj 1861(1 Pt B): 3581-3592. <https://doi.org/10.1016/j.bbagen.2016.06.020>

De Sousa-D'Auria C, Kacem R, Puech V, Tropis M, Leblon G, Houssin C, Daffé M (2003) New insights into the biogenesis of the cell envelope of *corynebacteria*: identification and functional characterization of five new mycoloyltransferase genes in *Corynebacterium glutamicum*. FEMS Microbiol Lett 224(1): 35-44. <https://doi.org/10.1016/s0378-1097(03)00396-3>

Devi A, Bajar S, Kour H, Kothari R, Pant D, Singh A (2022) Lignocellulosic Biomass Valorization for Bioethanol Production: a Circular Bioeconomy Approach. Bioenergy Res: 1-22. <https://doi.org/10.1007/s12155-022-10401-9>

Ding W, Si M, Zhang W, Zhang Y, Chen C, Zhang L, Lu Z, Chen S, Shen X (2015) Functional characterization of a vanillin dehydrogenase in *Corynebacterium glutamicum*. Sci Rep 5: 8044. <https://doi.org/10.1038/srep08044>

Evans R, O'Neill M, Pritzel A, Antropova N, Senior A W, Green T, Ídek A, Bates R, Blackwell S, Yim J (2021) Protein complex prediction with AlphaFold-Multimer. Cold Spring Harbor Laboratory.

Guo H, Zhao Y, Chang J S, Lee D J (2022) Inhibitor formation and detoxification during lignocellulose biorefinery: A review. Bioresour Technol 361: 127666. <https://doi.org/10.1016/j.biortech.2022.127666>

Kacem R, De Sousa-D'Auria C, Tropis M, Chami M, Gounon P, Leblon G, Houssin C, Daffé M (2004) Importance of mycoloyltransferases on the physiology of *Corynebacterium glutamicum*. Microbiology (Reading) 150(Pt 1): 73-84. <https://doi.org/10.1099/mic.0.26583-0>

Kamimura N, Takahashi K, Mori K, Araki T, Fujita M, Higuchi Y, Masai E (2017) Bacterial catabolism of lignin-derived aromatics: New findings in a recent decade: update on bacterial lignin catabolism. Environ Microbiol Rep 9(6): 679-705. <https://doi.org/10.1111/1758-2229.12597>

Lanéelle M A, Tropis M, Daffé M (2013) Current knowledge on mycolic acids in *Corynebacterium glutamicum* and their relevance for biotechnological processes. Appl Microbiol Biotechnol 97(23): 9923-9930. <https://doi.org/10.1007/s00253-013-5265-3>

Lin K, Han S, Zheng S (2022) Application of *Corynebacterium glutamicum* engineering display system in three generations of biorefinery. Microb Cell Fact 21(1): 14. <https://doi.org/10.1186/s12934-022-01741-4>

Liu Y, Long M, Yin Y, Si M, Zhang L, Lu Z, Wang Y, Shen X (2013) Physiological roles of mycothiol in detoxification and tolerance to multiple poisonous chemicals in *Corynebacterium glutamicum*. Arch Microbiol 195(6): 419-429. <https://doi.org/10.1007/s00203-013-0889-3>

Marchler-Bauer A, Bo Y, Han L, He J, Lanczycki C J, Lu S, Chitsaz F, Derbyshire M K, Geer R C, Gonzales N R, Gwadz M, Hurwitz D I, Lu F, Marchler G H, Song J S, Thanki N, Wang Z, Yamashita R A, Zhang D, Zheng C, Geer L Y, Bryant S H (2017) CDD/SPARCLE: functional classification of proteins via subfamily domain architectures. Nucleic Acids Res 45(D1): D200-d203. <https://doi.org/10.1093/nar/gkw1129>

Marrakchi H, Lanéelle M A, Daffé M (2014) Mycolic acids: structures, biosynthesis, and beyond. Chem Biol 21(1): 67-85. <https://doi.org/10.1016/j.chembiol.2013.11.011>

Puech V, Bayan N, Salim K, Leblon G, Daffé M (2000) Characterization of the in vivo acceptors of the mycoloyl residues transferred by the *corynebacterial* PS1 and the related *mycobacterial* antigens 85. Mol Microbiol 35(5): 1026-1041. <https://doi.org/10.1046/j.1365-2958.2000.01738.x>

Rahlwes K C, Sparks I L, Morita Y S (2019) Cell Walls and Membranes of *Actinobacteria*. Subcell Biochem 92: 417-469. <https://doi.org/10.1007/978-3-030-18768-2_13>

Rodriguez A, Meadows J A, Sun N, Simmons B A, Gladden J M (2021) Evaluation of bacterial hosts for conversion of lignin-derived p-coumaric acid to 4-vinylphenol. Microb Cell Fact 20(1): 181. <https://doi.org/10.1186/s12934-021-01670-8>

Shen X, Jiang C, Huang Y, Liu Z, Liu S (2005) Functional identification of novel genes involved in the glutathione-independent gentisate pathway in *Corynebacterium glutamicum*. Appl Environ Microbiol 71(7): 3442-3452. <https://doi.org/10.1128/aem.71.7.3442-3452.2005>

Shen X, Zhou N, Liu S (2012) Degradation and assimilation of aromatic compounds by *Corynebacterium glutamicum*: another potential for applications for this bacterium? Appl Microbiol Biotechnol 95(1): 77-89. <https://doi.org/10.1007/s00253-012-4139-4>

Si M, Zhao C, Burkinshaw B, Zhang B, Wei D, Wang Y, Dong T G, Shen X (2017) Manganese scavenging and oxidative stress response mediated by type VI secretion system in *Burkholderia thailandensis*. Proc Natl Acad Sci U S A 114(11): E2233-e2242. <https://doi.org/10.1073/pnas.1614902114>

Takeshita R, Ito H, Wachi M (2010) A role of the cspA gene encoding a mycolyltransferase in the growth under alkaline conditions of *Corynebacterium glutamicum*. Biosci Biotechnol Biochem 74(8): 1617-1623. <https://doi.org/10.1271/bbb.100214>

Tamura K, Stecher G, Kumar S (2021) MEGA11: Molecular Evolutionary Genetics Analysis Version 11. Mol Biol Evol 38(7): 3022-3027. <https://doi.org/10.1093/molbev/msab120>

Wang X, Khushk I, Xiao Y, Gao Q, Bao J (2018) Tolerance improvement of *Corynebacterium glutamicum* on lignocellulose derived inhibitors by adaptive evolution. Appl Microbiol Biotechnol 102(1): 377-388. <https://doi.org/10.1007/s00253-017-8627-4>

Wolf S, Becker J, Tsuge Y, Kawaguchi H, Kondo A, Marienhagen J, Bott M, Wendisch V F, Wittmann C (2021) Advances in metabolic engineering of *Corynebacterium glutamicum* to produce high-value active ingredients for food, feed, human health, and well-being. Essays Biochem 65(2): 197-212. <https://doi.org/10.1042/ebc20200134>

Zhao L, Sun Z F, Zhang C C, Nan J, Ren N Q, Lee D J, Chen C (2022) Advances in pretreatment of lignocellulosic biomass for bioenergy production: Challenges and perspectives. Bioresour Technol 343: 126123. <https://doi.org/10.1016/j.biortech.2021.126123>

Zhou P, Khushk I, Gao Q, Bao J (2019) Tolerance and transcriptional analysis of Corynebacterium glutamicum on biotransformation of toxic furaldehyde and benzaldehyde inhibitory compounds. J Ind Microbiol Biotechnol 46(7): 951-963. <https://doi.org/10.1007/s10295-019-02171-9>

## Figure Captions

**Fig. 1** *cgl2215* transcription in *C. glutamicum* in response to phenolic compounds and bioinformatics analysis of Cgl2215 protein*.* **A** qRT-PCR analysis of *cgl2215* transcription in *C. glutamicum* exposure to phenol (4 mM), ferulic acid (3 mM), or vanillin (3 mM) stresses. **B** Phylogenetic tree analysis of Cgl2215 and its homologs. **C** Multiple-sequence alignment analysis of Cgl2215 and its homologs.

**Fig. 2** Effect of *cgl2215* deletion on the tolerance to multiple environmental stresses in *C. glutamicum*. **A** Estimation of *C. glutamicum* tolerance to phenol (32 mM), ferulic acid (5 mM), or vanillin (20 mM) by LB agar-plate culture. **B** Survival rates of *C. glutamicum* strains after exposure to phenol (50 mM), ferulic acid (8 mM) or vanillin (50 mM) stresses for 30 min. **C** Survival rates of *C. glutamicum* strains after exposure to H_2_O_2_ (20 mM), ethanol (1.72 M), or heat (42°C) stresses for 30 min. The mean values and SD from at least three repeats are shown. **P* < 0.05; ***P* < 0.01; ****P* < 0.001; *****P* < 0.0001.

**Fig. 3** Cgl2215 interacting with MytA. **A** Screening of Cgl2215-interacting proteins by GST pull-down coupled with mass spectrometry. The target proteins captured by magnetic beads were separated by SDS-PAGE and silver stained. *C. glutamicum* cell lysates (lane 1), the captured proteins by beads coated with GST (lanes 2 and 4) or GST-Cgl2215 (lanes 3 and 5), purified GST protein (lane 6), and GST-Cgl2215 protein (lane 7). **B** GST pull-down to verify the interaction between MytA and Cgl2215. The lysate supernatants of *E. coli* strains expressing His_6_-MytA only or expressing His_6_-MytA with either GST-Cgl2215 or GST were individually loaded onto the GST-bind resin, and the resin was then washed to remove nonspecific-bounded proteins. The initial lysate (input) and retained proteins (pull-down) were analyzed by western blot against GST or His antibody. GST was as a negative control and GST-YezP was as an unrelated control. **C** Bacterial two-hybrid results to verify the interaction between MytA and Cgl2215. The *cgl2215* and *mytA* DNA fragments were cloned into bacterial two-hybrid system for colony color and enzymatic activity assay. T18-*mytA*/T25-*cgl2215*: *E. coli* BTH101 containing pKT18cm-*mytA* and pKT25m-*cgl2215*; T18/T25-*cgl2215*: *E. coli* BTH101 containing pKT18cm and pKT25m-*cgl2215*; T18-*mytA*/T25: *E. coli* BTH101 containing pKT18cm-*mytA* and pKT25m; T18/T25: *E. coli* BTH101 containing pKT18cm and pKT25m; T18-*zip*/T25-*zip*: *E. coli* BTH101 containing pKT25m-*zip* and pKT18cm-*zip.*

**Fig. 4** MytA involved in Cgl2215-mediated tolerance to phenolic compounds in *C. glutamicum*. **A** qRT-PCR analysis of *mytA* transcription in *C. glutamicum* in response to phenol (8 mM), ferulic acid (4 mM), vanillin (6 mM), H_2_O_2_ (20 mM), ethanol (1.72 M), or heat (42°C). **B** Effect of *mytA* deletion on the tolerance to phenol (50 mM), ferulic acid (8 mM), or vanillin (50 mM). **C** Survival rates of *C. glutamicum* Δ*cgl2215*Δ*mytA*(vector) strain and its different complemented stains after exposure to phenol (50 mM) stress for 30 min. The mean values and SD from at least three repeats are shown. ***P* < 0.01; ****P* < 0.001; *****P* < 0.0001.

**Fig. 5** Effect of Cgl2215 on the *in vitro* esterase activity and cell envelope of *C. glutamicum*. **A** Prediction of the MytA and Cgl2215 complex by the AlphaFold-Multimer. MytA and Cgl2215 are represented as cartoons in cyan and green, and the possible interaction regions on two chains are indicated by blue and rose. **B** *In vitro* esterase activity assay of the purified MytA proteins with the addition of the purified GST or GST-Cgl2215 proteins. The mean values and SD from at least two repeats are shown. *****P* < 0.0001. **C** TEMs of *C. glutamicum* strains. PM, plasma membrane; EDL, electron-dense layer; OL, outer layer; ETL, electron-transparent layer.

## Supplementary Information

**Additional file 1: Fig. S1** Growth curve of *C. glutamicum* wild-type strain RES167 in MS medium supplemented with phenol (4 mM), ferulic acid (3 mM), or vanillin (3 mM). **Fig. S2** Growth curves of *C. glutamicum* WT(vector), Δ*cgl2215*(vector) and Δ*cgl2215*(*cgl2215*) strains in LB broth. **Table S1** Information of Cgl2215 homologs from non-redundant protein database. **Table S2** Bacterial strains and plasmids used in this study. **Table S3** Primers used in this study.
